# Multi-Organ Damage in Human Dipeptidyl Peptidase 4 Transgenic Mice Infected with Middle East Respiratory Syndrome-Coronavirus

**DOI:** 10.1371/journal.pone.0145561

**Published:** 2015-12-23

**Authors:** Guangyu Zhao, Yuting Jiang, Hongjie Qiu, Tongtong Gao, Yang Zeng, Yan Guo, Hong Yu, Junfeng Li, Zhihua Kou, Lanying Du, Wenjie Tan, Shibo Jiang, Shihui Sun, Yusen Zhou

**Affiliations:** 1 State Key Laboratory of Pathogen and Biosecurity, Beijing Institute of Microbiology and Epidemiology, Beijing, 100071, China; 2 Lindsley F. Kimball Research Institute, New York Blood Center, New York, New York, 10065, United States of America; 3 Key Laboratory of Medical Molecular Virology of Ministries of Education and Health, Shanghai Medical College, Fudan University, Shanghai, 200433, China; 4 Key Laboratory of Medical Virology, Ministry of Health, National Institute for Viral Disease Control and Prevention, China CDC, Beijing, 102206, China; Deutsches Primatenzentrum GmbH - Leibniz-Institut fur Primatenforschung, GERMANY

## Abstract

The Middle East Respiratory Syndrome Coronavirus (MERS-CoV) causes severe acute respiratory failure and considerable extrapumonary organ dysfuction with substantial high mortality. For the limited number of autopsy reports, small animal models are urgently needed to study the mechanisms of MERS-CoV infection and pathogenesis of the disease and to evaluate the efficacy of therapeutics against MERS-CoV infection. In this study, we developed a transgenic mouse model globally expressing codon-optimized human dipeptidyl peptidase 4 (hDPP4), the receptor for MERS-CoV. After intranasal inoculation with MERS-CoV, the mice rapidly developed severe pneumonia and multi-organ damage, with viral replication being detected in the lungs on day 5 and in the lungs, kidneys and brains on day 9 post-infection. In addition, the mice exhibited systemic inflammation with mild to severe pneumonia accompanied by the injury of liver, kidney and spleen with neutrophil and macrophage infiltration. Importantly, the mice exhibited symptoms of paralysis with high viral burden and viral positive neurons on day 9. Taken together, this study characterizes the tropism of MERS-CoV upon infection. Importantly, this hDPP4-expressing transgenic mouse model will be applicable for studying the pathogenesis of MERS-CoV infection and investigating the efficacy of vaccines and antiviral agents designed to combat MERS-CoV infection.

## Introduction

The highly pathogenic Middle East respiratory syndrome-coronavirus (MERS-CoV) was first documented in the Middle East in 2012. As of November 13, 2015, a total of 1618 cases had been reported by the World Health Organization, including 579 fatalities (http://www.who.int/csr/don/13-november-2015-mers-saudi-arabia/en/). The mortality rate of MERS-CoV is as high as 35%, far higher than that of the SARS coronavirus (8–10%) [1]. Although milder and asymptomatic presentation does occur, a portion of patients develop severe respiratory disease identified as viral pneumonia and acute respiratory distress syndrome (ARDS) accompanied by leucopenia and lymphopenia, similar to patients infected with SARS-CoV. In addition, at least one-third of patients exhibit gastrointestinal symptoms and in some severe cases, renal failure develops concurrently with MERS-CoV infection [2, 3]. More recently, severe neurologic syndrome was also reported in critically cases [4]. Although the limited clinical data available indicate that systemic infection can occur, the pathological mechanism of multi-organ damage caused by MERS-CoV infection is not well understood.

Receptor specificity appears to be a key factor in the tropism of MERS-CoV [5], and dipeptidyl peptidase 4 (DPP4) has been identified as the functional cellular receptor for MERS-CoV [6]. In humans, DPP4 is constitutively expressed in epithelial cells in the liver, intestine, prostate, kidneys and in activated leukocytes [7]. MERS-CoV may infect human tracheobronchial epithelium, primary renal epithelium, alveolar adenocarcinoma, liver cells (Huh-7) and bronchiolar epithelial cells [8]. Because they do not express human DPP4, mice, hamsters and ferrets cannot be infected with MERS-CoV [9–12]. However, rhesus macaques can be infected with MERS-CoV. Following infection, rhesus macaques develop transient clinical symptoms with histopathologically evident multifocal mild-to-moderate interstitial pneumonia on day 3 post-infection [13]. Common marmosets infected with high doses of MERS-CoV develop more severe disease than rhesus macaques, with sometimes fatal pneumonia [14]. In MERS-CoV-infected common marmosets, clinical disease was more severe and viral loads in the lungs were higher than in the rhesus macaque lungs. However, while viral RNA was detected in kidney samples from five out of seven common marmosets, no histological abnormalities were observed. Furthermore, the ethics, higher expense and sophisticated operation limited their application for the studies on the pathogenesis of the disease and efficient evaluation of vaccines or drugs to treat MERS-CoV infection.

Stanley Perlman’s research group developed an Ad5-hDPP4-transduced mouse able to maintain MERS-CoV infection [15]. Although this model may be used efficiently for evaluating the efficacy of vaccines and drugs, it is still limited in its ability to recapitulate the pathogenesis of the disease for its transient and self-limited viral infection. To develop a more susceptible, stable and efficient model, Chien-Te K. Tseng’s group successfully established a transgenic mice model stably expressing hDPP4 that exhibited progressive pneumonia and lethal outcome after MERS-CoV infection [16]. In the model, although no tissue damage was observed in the brain, viral replication was reported. More recently, another hDPP4 mouse model was developed by replacing 79 kb mouse DPP4 genomic DNA with that encoding 82 kb human genomic DPP4 using the VelociGene techonology. Although no mortality or clinical signs of disease was observed up to day 4, robust viral replication and pathology in the lungs were detected. No specific MERS-CoV RNA and information was detected in brain [17]. In this study, we generated a transgenic mouse model also that globally expresses codon-optimized-hDPP4. This MERS-CoV infected optimized-hDPP4 transgenic mice developed severe pneumonia with multi-organ injury including liver, kidney and brain damages.

## Methods

### Ethics statement

All procedures involving animals were approved by the Laboratory Animal Center, State Key Laboratory of Pathogen and Biosecurity, Beijing Institute of Microbiology and Epidemiology IACUC’s (The permit number is BIME 2014–0017). Animal studies were carried out in strict accordance with the recommendations in the Guide for the Care and Use of Laboratory Animals. All experimental operations to mice were performed under sodium pentobarbital anesthesia, and mice were euthanized with overdose inhalational carbon dioxide. All efforts were made to minimize suffering of animals.

### Determination of hDPP4 expression in cell lines

**Fig 1:**
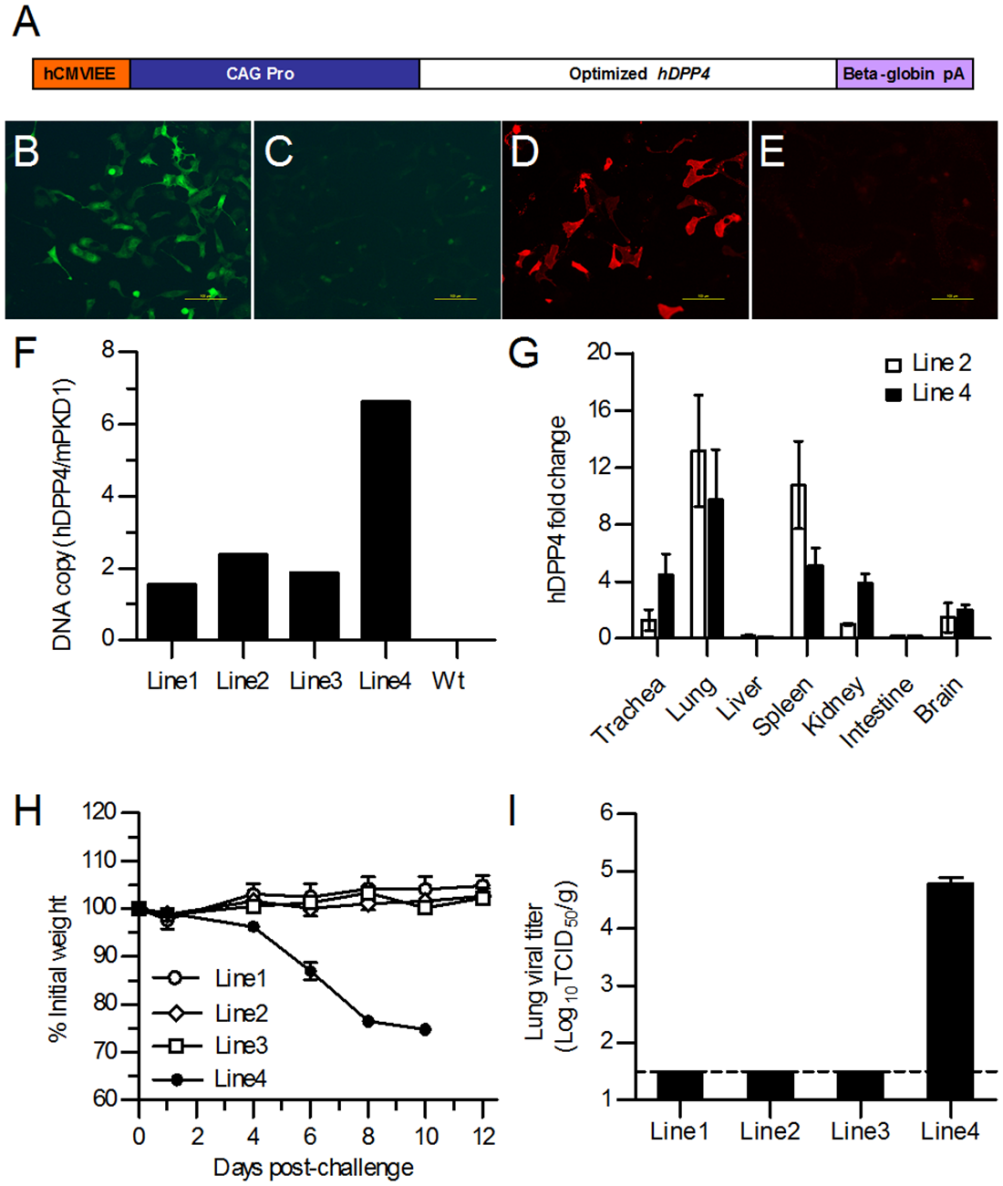
Generation and characterization of hDPP4 transgenic mice. (A) Schematic diagram of the optimized hDPP4 expression vector cassette. The optimized hDPP4 was cloned into a pCAGGS plasmid in which hDPP4 expression was driven by the CAG promoter. hDPP4 expression was confirmed *in vitro* by transfection of Cos-7 cells with the pCAGGS-hDPP4 (B) or pCAGGS (C) plasmids and detected by direct immunofluorescence assay with FITC-labeled anti-human CD26–Fluorescein antibody. Confirmation of the binding between DPP4 and MERS-CoV RBD was achieved by transfecting Cos-7 cells with pCAGGS-hDPP4 (D) or pCAGGS (E) plasmid followed by indirect immunofluorescence assay with MERS-RBD-Fc protein and DyLight 549-conjugated goat anti-human IgG antibody. (F) Determination of the copy numbers of hDPP4 cDNA in four transgenic founder lines by qPCR. (G) Expression of hDPP4 mRNA in the indicated tissues of transgenic mice in two founder lines as determined by qRT-PCR. Results are mean±SEM (*n* = 3). (H) Four lines of hDPP4 transgenic mice were infected with MERS-CoV and monitored for body weight changes. Results are mean±SEM (*n* = 6). (I) Lung viral titer at day 5 postinfection was determined for four lines of hDPP4 transgenic mice. The data are expressed as mean±SEM (*n* = 3). The dotted line indicates the limit of detection.

Considering the species differences, the hDPP4-coding sequence was codon-optimized and synthesized (GenScript, Nanjing, China) according to the mRNA sequence (Accession number NM_001935.3) and cloned into the multiple cloning site (MCS) of a pCAGGS plasmid, leading to strong expression of the gene. The recombinant plasmid pCAGGS-hDPP4 contained the hCMV IE Enhancer (hCMVIEE), the CAG promoter (chicken β-actin promoter/intron), a codon-optimized open reading frame (ORF) of hDPP4 and rabbit β-globin polyA (Fig 1A). Expression of the codon-optimized hDPP4 was achieved by transfection of the pCAGGS-hDPP4 plasmid into Cos-7 cells, followed by confirmation of expression using anti-human CD26 monoclonal antibody (R&D Systems, Minneapolis, MN, USA). To confirm the binding of hDPP4 to the MERS-CoV receptor-binding domain (RBD), transfected Cos-7 cells were incubated with a recombinant protein expressing the RBD of MERS-CoV fused to human IgG Fc (RBD-Fc) [18], followed by addition of DyLight 549-conjugated goat anti-human IgG to detect binding.

### Determination of hDPP4 copy number and transcription levels in transgenic mice

The purified 5861bp fragment generated from ApaL1 digestion of pCAGGS-hDPP4 was used as the transgene for injection into the pronuclei of fertilized F1 (C57BL/6 × C57BL/6) mouse eggs to generate transgenic embryos. The mice used in this studywere backcrossed two to three times onto a C57BL/6 background. All animal studies were performed in strict accordance with the recommendations outlined in the Guide for the Care and Use of Laboratory Animals.

Genomic DNA from each transgenic founder line and from wild-type C57BL/6 mice was isolated from the liver tissue using DNAzol reagent (Qiagen, Valencia, CA, USA) according to the manufacturer`s instructions. The hDPP4 DNA copy numbers were determined by quantitative PCR using Power SYBR^®^ Green PCR Master Mix (Life Technologies, Carlsbad, CA, USA). Total hDPP4 DNA was normalized using a single-copy reference gene (PKD1) as an endogenous control. The primers specific for hDPP4 were forward 5’ CTACAGCTCCTGGGCAACGTGCTG 3’ and reverse 5’ AGATGTCGTAACTGGCGGTGTAAG 3’. The primers specific for mPKD1 were forward 5’ GGCTGCTGAGCGTCTGGTA 3’ and reverse 5’ CCAGGTCCTGCGTGTCTGA 3’.

To detect the expression of optimized hDPP4 in the tissues of our transgenic mice model, samples were harvested and stored immediately in RNA*later* RNA stabilization reagent (Qiagen, Valencia, CA, USA) until total RNA was extracted and purified using an RNeasy Extraction kit (Qiagen, Valencia, CA, USA). For each sample, 2 μg of total RNA was used as template for first-strand cDNA synthesis. The resulting cDNA was subjected to quantitative PCR using Power SYBR^®^ Green PCR Master Mix (Life Technologies, Carlsbad, CA, USA) to determine the relative abundances of hDPP4 in the tissues. The forward and reverse primers for hDPP4 amplicons were as the same as those noted above. The relative amount of hDPP4 in different tissues was obtained by normalizing mRNA expression to that of the endogenous control gene GAPDH [19].

### Infection of hDPP4 transgenic mice with MERS-CoV and sample collection

MERS-CoV (HCoV-EMC/2012 strain) was propagated and titered on Vero cells in an approved biosafety level 3 laboratory. Following intraperitoneal anesthetization with sodium pentobarbital (5 mg/kg of body weight), mice under virus infection were intranasally inoculated with MERS-CoV (10^4.3^ TCID_50_) in 20 μl Dulbecco’s modified Eagle’s medium (DMEM), and mice in sham group were treated with the same volume of DMEM. Mice were monitored for weight loss and survival for 12 days. We have taken special precautions and followed standard guidelines outlined in the Guide for the Care and Use of Laboratory Animals to ensure that ample food and water as well as sanitary cage conditions with enrichment devices were present in order to maximize the animal’s comfort. Humane endpoints were used during the survival experiments. After virus infection, mice were closely monitored by daily observation and weighing of trained animal caretaker, and the monitoring will raise to twice per day once mice lost more than 10% of their initial body weight, or exhibited lethargy, ruffled hair coat, or hunched posture. Animals were deemed gravely ill and were euthanized by overdose inhalation of carbon dioxide if they lost more than 25% of their weight, or lost ability to ambulate and access food or water. Sera were collected on day 5 and inactivated at 56°C for 30 min before the levels of cytokines and chemokines were measured. To assess viral replication and histopathologic damage following MERS-CoV infection, mice were euthanized with overdose inhalational carbon dioxide, and tissues included lungs, kidneys, livers, spleens, intestines and brains were harvested on indicated time points.

### Detection of MERS-CoV RNA in hDPP4 transgenic mouse tissues

The viral RNA copies in tissues were determined by qRT-PCR according to the protocol described elsewhere [20]. Briefly, total RNA was extracted from 20mg of collected tissues using RNeasy Extraction Kits (Qiagen, Valencia, CA, USA) following the manufacturer’s instructions. MERS-CoV RNA was quantified in a 25 μl mixture containing 5 μl RNA using the Invitrogen SuperScript^™^ III One-Step RT-PCR system with Platinum^®^ Taq (Life Technologies, Carlsbad, CA, USA). The primers and probes specific for the upE envelope gene of the MERS-CoV virus were as follows: forward 5’ GCAACGCGCGATTCAGTT 3’; reverse 5’ GCCTCTACACGGGACCCATA 3’; fluorescence probe /56-FAM/ CTCTTCACATAATCGCCCCGAGCTCG CG/36-TAMSp/ [21]. Mouse GAPDH was used as a housekeeping gene control. A standard curve was generated for PCR reaction using 10-10^7^copies of quantified RNA transcripts to calculate copy numbers for each reaction. The results were considered positive at C_T_ values below 35 for primer and probe set, and were calculated as viral RNA copies per gram of tissues.

### Viral titer in tissue

The tissues of infected mice were harvested aseptically at indicated time points and homogenized in minimal essential medium (MEM) plus antibiotics to produce 10% (w/v) suspensions. Tissue homogenates were centrifuged and titered on the monolayer of Vero cells. The cytopathic effects (CPE) were daily observed under phase-contrast microscropy for 3 days. The viral titer was determined as TCID_50_ by CPE-based assay, and calculated using the Reed and Muench method. The viral titer in tissue was expressed as Log_10_ TCID_50_/g of tissue.

### Histopathology and immunohistochemistry

Collected tissue samples were immediately fixed in 10% neutral buffered formalin, sectioned at 4 μm thickness, and stained with hematoxylin and eosin (H&E) to examine histopathology as described previously [22]. The expression of virus antigens and infiltration of neutrophils and macrophages in organs after MERS-CoV infection was detected by immunohistochemistry. Briefly, formalin-fixed, paraffin-embedded lung sections were deparaffinized and hydrated using graded alcohols. Expression of virus antigen and inflammatory cell infiltration was assessed using rabbit polyclonal antibody to MERS-CoV nucleoprotein (NP) (Sino Biological Inc., Beijing, China), neutrophil marker NIMP-14, (Santa Cruz Biotechnology, Paso Robles, CA, USA) and CD68 (Abcam, Cambridge, MA, USA). The reaction was detected using a standard streptavidin-biotin detection system (Beijing Zhongshan Biotechnology Co., Ltd., Beijing, China) and visualized using DAB with hematoxylin counterstaining.

### Detection of inflammatory cytokines and chemokines

Cytokines and chemokines in mouse sera were quantified using a commercial Milliplex Mouse Cytokine/Chemokine Magnetic Panel kit (Merck Millipore, USA.). A panel of inflammatory cytokines and chemokines (IFN-γ, IP-10, IL-1β, IL-17, IL-6, TNF-α, GM-CSF, KC, MCP-1, MIP-1ɑ, MIP-1β and RANTES) was measured according to the manufacturer’s protocols.

### Statistics

Statistical analyses were performed using GraphPad Prism version 5.01. To compare the groups in terms of hDPP4 copies, inflammatory cytokine/chemokine levels, and tissue viral RNA copies, Student’s *t* test with Welch’s correction was used. The significance between survival curves was analyzed by Kaplan-Meier survival analysis with log-rank test. *p* values lower than 0.05 were considered statistically significant.

## Results

### Generation and characterization of transgenic mice expressing hDPP4

DPP4 is constitutively expressed in human parenchyma cells in tissues including the liver, intestines and kidneys as well as in T and B cells [7]. We developed transgenic mice in which hDPP4 expression was driven by the chicken β-actin promotor in the pCAGGS plasmid (Fig 1A). Expression of hDPP4 *in vitro* was achieved by transfection of the pCAGGS-hDPP4 plasmid into Cos-7 cells and detected by immunofluorescence analysis. The results showed that hDPP4 was expressed in Cos-7 cells and that it effectively bound MERS-CoV (Fig 1B–1E). After microinjection of linearized DNA, founder lines that transferred the hDPP4 gene to their progeny were established. The levels of transgenic DNA in the founder lines ranged from 2 to 6 copies per genome, as determined by qPCR (Fig 1F). In addition, the mRNA levels of hDPP4 in the trachea, lung, liver, spleen, kidney, intestine and brain in two of the founder lines were measured by qRT-PCR. The data showed that hDPP4 expression was higher in the trachea, lung, spleen, kidney and brain in both lines, and the expressions were relative higher in trachea and kidney in line 4 compared with that in line 2 (Fig 1G). In order to determine the effective infection of MERS-CoV in different lines of transgenic mice, 6 mice in each line were infected with MERS-CoV and the changes of body weight were monitored. The results showed that all mice in line 1, 2 and 3 survived with no significant changes in body weight but all mice in line 4 died by day 10 following MERS-CoV inoculation (Fig 1H). In order to confirm the virus replications in transgenic mice, three mice of each line were sacrificed on day 5 after MERS-CoV infection for the detection of viral titer and viral antigen expression in lungs. As expected, compared with high level of lung viral titers in line 4 mice, no viral titer were detected (Fig 1I) in lungs of line 1, 2 and 3 mice, which also confirmed by the negative results of viral antigen expression detected by immunohistochemistry in lung (data not shown).

### Multi-organ tissue damage in hDPP4 transgenic mice infected with MERS-CoV

**Fig 2:**
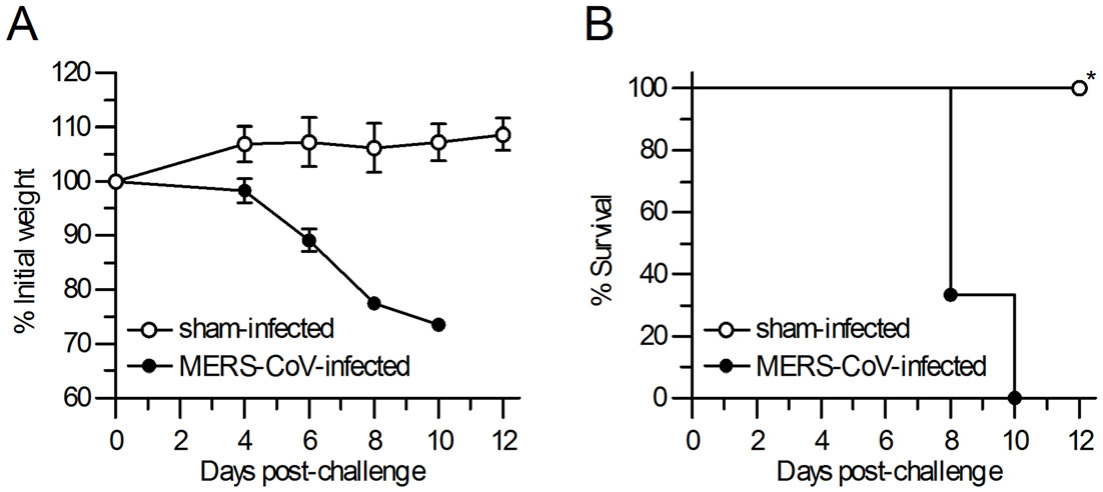
MERS-CoV infection resulted in lethal disease in hDPP4 transgenic mice. Transgenic mice were infected intranasally with MERS-CoV and monitored for 12 days. (A) Body weight changes of MERS-CoV- and sham-infected mice. Results are mean±SEM (*n* = 3) on indicated time post-infection. (B) Survival curves of mice in MERS-CoV- and sham-infected mice (*n* = 3). * *p* < 0.05.

Wild-type mice, such as BALB/c, 129/SvEv and STAT1 mice, are not permissive to MERS-CoV infection, and Syrian hamsters do not support replication of MERS-CoV [9–12]. Macaques have been confirmed to develop mild to marked interstitial pneumonia upon MERS-CoV infection [23], but fail to progress to the extent observed in human subjects. Marmoset model, which has the same interaction characteristic between its DPP4 and MERS-CoV spike protein, was partially lethal and developed a progressive severe pneumonia after MERS-CoV infection [14]. In this study, we found that hDPP4 transgenic mice infected with MERS-CoV exhibited decreased activity and significant weight loss from day 6 after infection, and all of the infected mice died by day 10 (Fig 2A and 2B). Importantly, symptoms of neural defects with paralysis were observed on day 9 in the infected transgenic mice.

**Fig 3:**
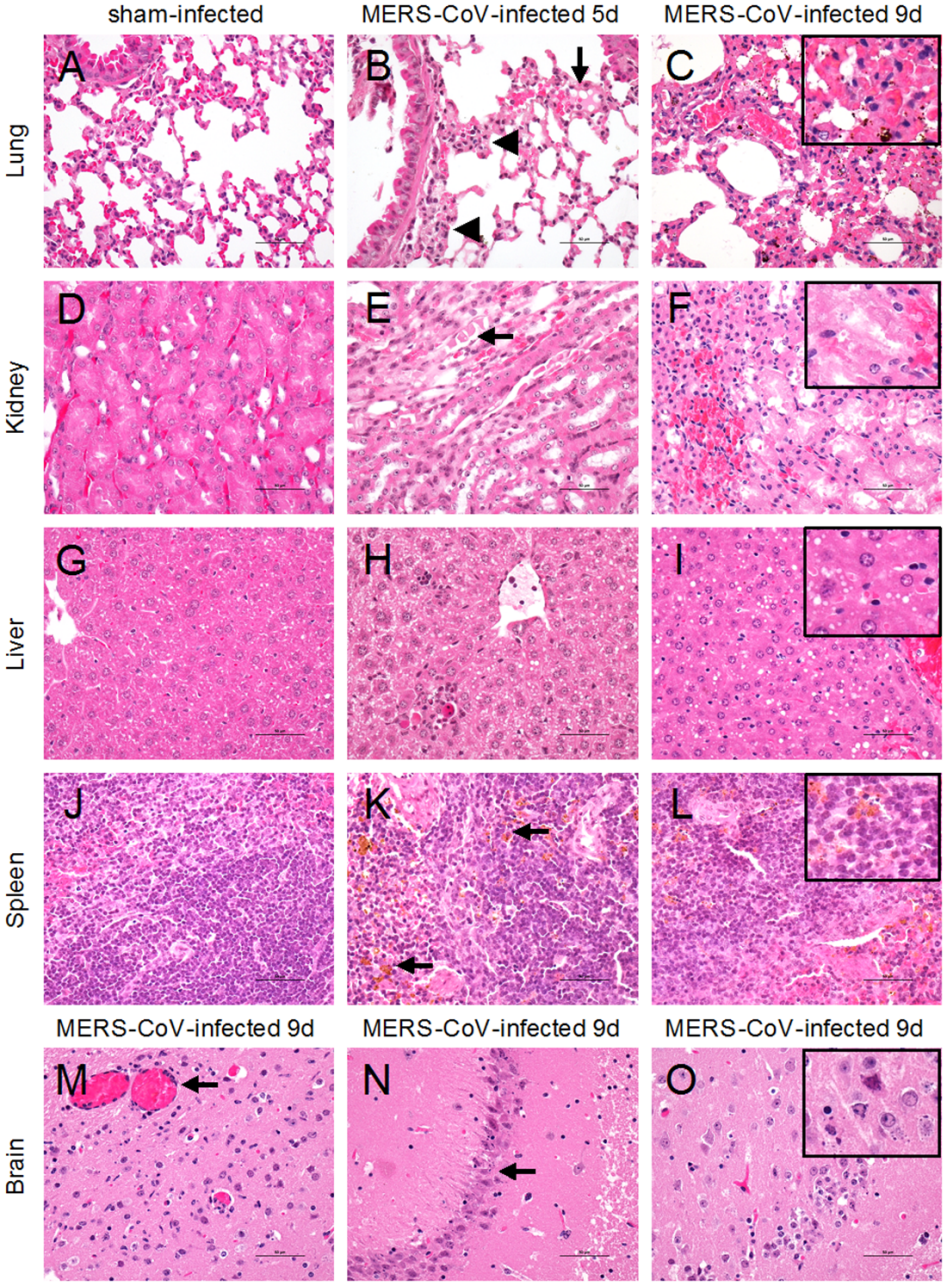
Histopathological analysis of hDPP4 transgenic mice after MERS-CoV infection. The hDPP4 transgenic mice were infected intranasally with MERS-CoV. Lungs, kidneys, liver, spleen and brain were collected on day 5 or day 9 after MERS-CoV infection, fixed in neutral formaldehyde solution and stained with hematoxylin and eosin. Histopathological analysis of hDPP4 transgenic mice was performed using sham-infected mice as controls (A, D, G, J). (B-C) In the lungs, mild inflammation was observed, with inflammatory cell infiltration (arrowheads) and focal hemorrhage and exudation (arrow) on day 5. Damage was more severe on day 9, with increased inflammatory cell infiltration, focal hemorrhage (inset) and exudation. (E-F) In the kidney, MERS-CoV-infected hDPP4 transgenic mice showed focal interstitial inflammation with inflammatory cell infiltration in the interstitium and exudates in renal tubules (arrow) on day 5. On day 9, degeneration and necrosis in the renal tubular epithelial cells (inset) and focal hemorrhage were observed. (H-I) In the liver, MERS-CoV-infected hDPP4 transgenic mice showed scattered hepatocyte necrosis and numerous activated kupffer cells and infiltrated macrophages in the hepatic sinusoid on day 5. Fatty change of hepatocytes (inset) was observed in the liver on day 9. (K-L) In the spleen, necrotic splenic cells and increased reticulum cells in the red pulp with significant amounts of hemosiderin deposition were observed in hDPP4 transgenic mice on days 5 and 9 (arrow and inset). (M-O) Neurological damage with perivascular cuffs (M) and neuronal cell necrosis in the cerebral cortex (N) including damaged neurons (inset) in the hippocampus (O) was observed in the brains of hDPP4 transgenic mice infected with MERS-CoV. (*n* = 2, scale bars = 50 μm).

Compared to the sham-infected group (Fig 3A, 3D, 3G and 3J), the histopathological analysis of the tissues in transgenic mice on days 5 and 9 after MERS-CoV infection showed the presence of inflammatory tissue damage in the kidney, liver and spleen, with mild inflammatory responses in the lungs but no significant changes in the intestines. On day 5 after inoculation, the hDPP4 transgenic mice exhibited mild inflammation in the lungs with focal exudation and hemorrhage (Fig 3B), and by day 9, the damage was more severe, with evidence of diffused alveolar damage, alveolar septal thickening, hemorrhage and activated macrophage infiltration (Fig 3C). In the kidneys, mild interstitial inflammation with inflammatory cell infiltration was observed in the interstitium and exudates of renal tubules on day 5 (Fig 3E). By day 9, more renal tubular epithelial cells were injured, with evidence of focal hemorrhage in the renal interstitium (Fig 3F). In the liver, mild to moderate liver damage was seen in the transgenic mice infected with MERS-CoV on day 5, including scattered hepatocyte necrosis and numerous activated kupffer cells and macrophage infiltrates in hepatic sinusoid (Fig 3H), and on day 9, fatty change in hepatocytes with less hepatocyte necrosis was observed (Fig 3I). In spleens, necrosis of splenic cells and increased reticulum cells in red pulp with significant hemosiderin deposition was observed on both day 5 and day 9 (Fig 3K and 3L). Typical characteristics of viral encephalitis were observed in the brain at day 9, with perivascular cuffs (Fig 3M) and neuronal cell necrosis in the cerebral cortex (Fig 3N), including hippocampal neurons (Fig 3O). There was no apparent damage in the intestines after MERS-CoV infection on day 5 or day 9 (data not shown).

### Determination of virus antigen expression and viral replication in hDPP4 transgenic mice infected with MERS-CoV

**Fig 4:**
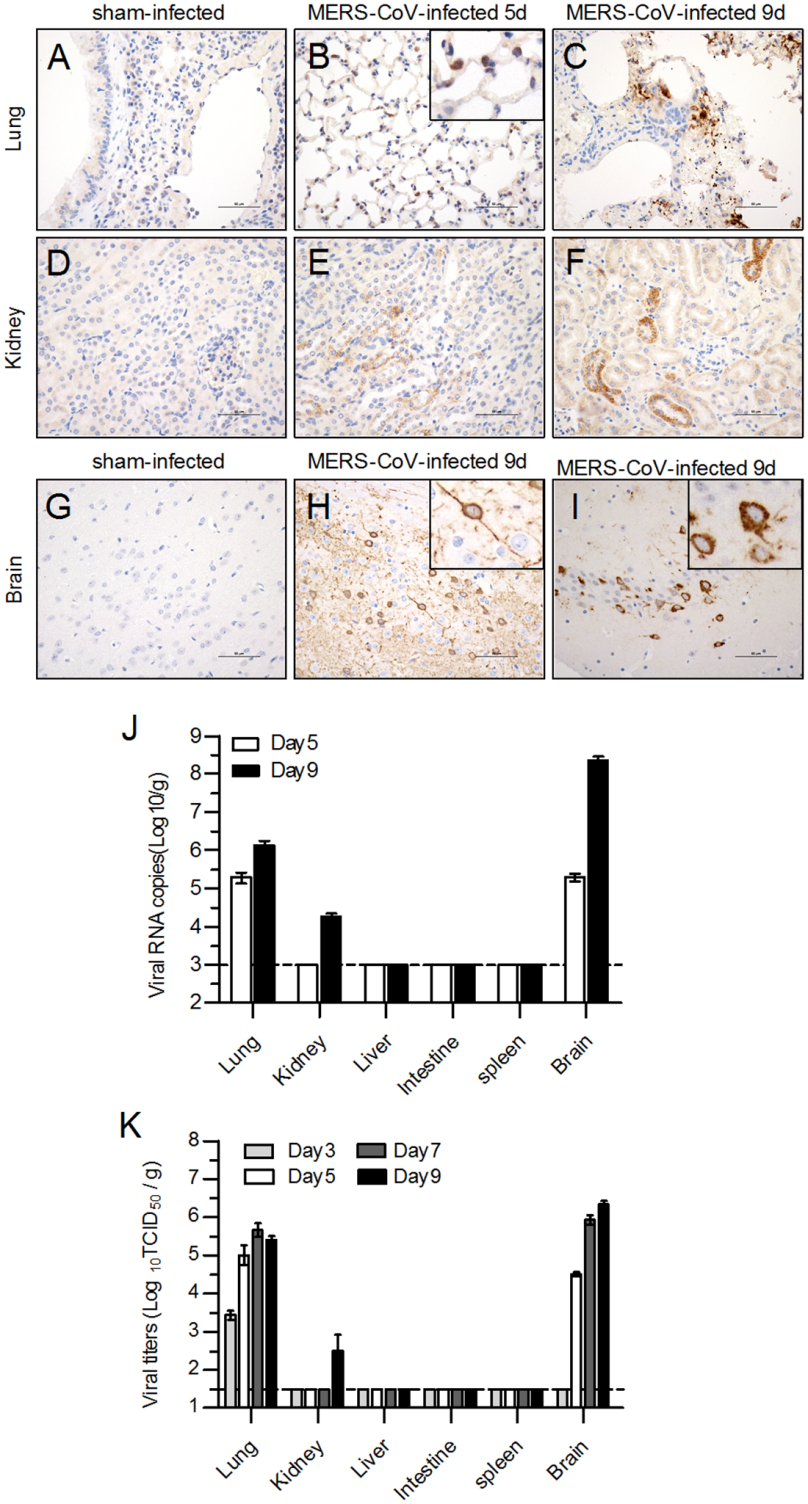
Detection of virus antigen expression and viral replications in the tissues of hDPP4 transgenic mice infected with MERS-CoV. Transgenic mice were infected intranasally with MERS-CoV and sacrificed to access the expression of viral antigens by immunohistochemical staining (*n* = 2). No viral antigens were detected in the sham-infected mice (A, D, G), but viral antigens were detected in type I and type II pneumocytes and infiltrated macrophages in the lungs (B-C), renal tubular epithelial cells in the kidneys (E-F), and neuron cell bodies as well as in dendrites and axons in the brain (H-I), including the hippocampus (I). (J) Viral load was detected by qRT-PCR and expressed as viral RNA copies/g of tissues. The detection limit shown as dotted line is 1×10^3^ copies/g. Results are mean±SEM (*n* = 2). (K) Viral titers in tissues of infected hDPP4 transgenic mice were determined by CPE-based assay and expressed as Log_10_TCID_50_/g of tissues. Results are mean±SEM (*n* = 5). The dotted line indicates the detection limit.

To evaluate MERS-CoV infection in the hDPP4 transgenic mice, distribution of the viral antigen NP protein was assessed in mouse tissues by immunohistochemical staining. The results showed that type I and type II pneumocytes in the lungs and renal tubular epithelial cells in the kidneys expressed viral antigen on day 5 after MERS-CoV infection and that the expression was more abundant on day 9, and no viral antigen was detected in sham-infected group (Fig 4A–4F). Strong viral antigen expression was evident not only in neuronal cell bodies, but also in dendrites, axons and some microglia in the cerebral cortex (Fig 4H) including the hippocampus (Fig 4I). No viral antigen expression was observed in the brains of mice in the sham-infected group (Fig 4G). Viral RNA copies in lung and brain were detectable on day 5 postinfection and increased to a higher level on day 9 (Fig 4J). Lower level of viral RNA copies can be also detected in kidney collected on day 9. In order to further confirm the effective replication of MERS-CoV, the dynamic changes of viral titer in organs were detected. The results showed that on day 3 after infection, only lower viral titer was detected in lung and not detectable in other organs. However, on day 5, viral titer was also detected in brain. On day 7 and day 9, increased viral titers were detected in both lung and brain, and lower level viral titer in kidney was detectable (Fig 4K). These results indicate that the transgenic mice had been successfully infected and that MERS-CoV exhibited cell and tissue tropism especially for pneumocytes and neurons. Furthermore, synapses may be one of the structures by which viruses diffuse through the brain after MERS-CoV infection.

### Increased inflammatory response in MERS-CoV-infected hDPP4 transgenic mice

**Fig 5:**
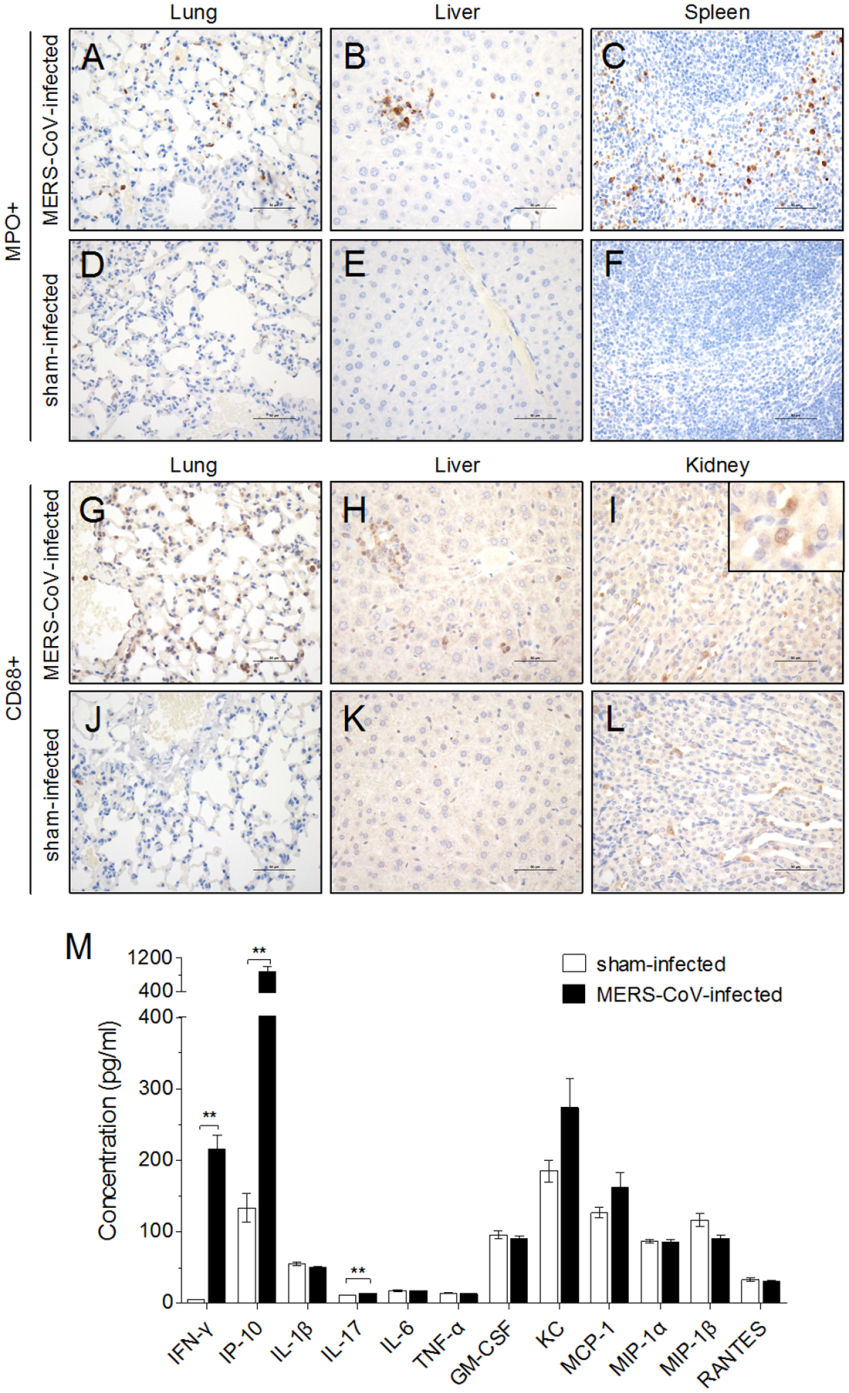
Detection of inflammatory cell infiltration and proinflammatory cytokines in the serum of hDPP4 transgenic mice infected with MERS-CoV. Neutrophil infiltration in the lungs, liver and spleen was evaluated 5 days after MERS-CoV infection (A-C) or sham infection (D-F). Macrophage infiltration in the lung, liver and kidney was detected 5 days after MERS-CoV (G-I) or sham infection (J-L) (*n* = 2). (M) Sera from the two groups of mice were collected on day 5 and assayed for levels IFN-γ, IP-10, IL-1β, IL-17, IL-6, TNF-α, GM-CSF, KC, MCP-1, MIP-1ɑ, MIP-1β and RANTES. Results are mean±SEM (*n* = 4). * *p* < 0.05.

To date, only limited data on the immune response of MERS patients are available [24]. Because of the clinical similarity between the symptoms of MERS-CoV and those of patients infected with SARS-CoV [1], the aberrant immune response may be related to the pathogenesis of MERS-CoV infection. In our mouse model, an aberrant inflammatory response was confirmed by infiltration of neutrophils in the lung, liver and spleen (Fig 5A–5F) and macrophages in the lung, liver and kidney (Fig 5G–5L). In addition, deposition of hemosiderin in the spleen (Fig 3K and 3L) indicated an increase in activated macrophages, and a systemic inflammatory response after virus infection was demonstrated by a significant increase in cytokines such as IFN-γ, IP-10, and IL-17 in the serum on day 5 after MERS-CoV infection (Fig 5M).

## Discussion

Although several species, such as rhesus macaques and common marmosets are reported to be sensitive to MERS-CoV infection and to develop mild to marked broncho-interstitial pneumonia, small animal models are urgently needed to study the pathogenesis of this disease and evaluate the effects of vaccines and antiviral agents. A MERS-CoV-infection mouse model was generated by transducing mice with a recombinant non-replicating adenovirus expressing hDPP4; however, while the mice exhibit viral antigen expression and progress to interstitial pneumonia, there is no mortality after virus infection [15]. Although a transgenic mouse model expressing human DPP4 was also established, and its immune response was studied after infection with MERS-CoV [16], the transgenic mice in the study died on day 6 with only progressive pneumonia and mild perivascular cuffing in brain, and no neurological disorder or other multi-organ damage was observed. Different from the aforementioned transgenic mice, our hDPP4 transgenic mice experienced a longer incubation period post-infection and developed progressive pneumonia and neurological disorders accompanied by histological damage to the lungs, brain, spleen and liver, which more closely resembles the clinical cases.

The results from this study indicate that MERS-CoV infection was lethal in hDPP4 transgenic mice with higher transgene copy number, while lines with lower levels of hDPP4 expressed especially in trachea had no significant clinical symptoms (data not shown), suggesting that the copy number of the transgene was closely related to the efficiency of MERS-CoV infection. Although the expression level of optimized hDPP4 was not highest in the brains, the tissues had higher viral titers following MERS-CoV infection, revealing the tropism of MERS-CoV for both lungs and brains, tissues in which pneumocytes and neurons may be the main target cells (Fig 4). However, MERS-CoV infection in brain via blood or olfactory nerves needs to be further studied due to the strong expression of viral antigens in dendrites and axons.

Although few autopsy reports exist on fatal MERS-CoV cases and atypical pneumonia and respiratory failure are suspected the causes of death [24], other types of extrapulmonary organ dysfunction have also been documented in MERS critically ill patients with abnormal clinical manifestations [25–27]. For example, acute renal failure was described in a number of MERS cases [28–32]. In addition, renal epithelial cells may produce almost 1000-fold more infectious MERS-CoV progeny than bronchial epithelial cells [33]. More recently, severe neurologic syndrome including altered level of consciousness from confusion to coma, ataxia and focal motor deficit was also reported in three critically cases by Balkhy H et al [4]. According to the neurologic manifestations and distinct imaging patterns, an important question was raised on the pathogenic mechanisms that underlie the occurrence of neurologic injury in patients. It has been studied that corona virus such as SARS-CoV are generally known for causing respiratory illness, and the strong tropism of SARS-CoV to CNS and causing neuronal injury had also been demonstrated both in clinical and experimental studies [34–38], which suggested the possible relations of MERS-CoV infection with brain injury. As to the extensive expression of hDPP4 in the lung, kidney, placenta, liver, skeletal muscle, brain, endothelium, pancreasour and T cell in human, our globally expressed hDPP4 transgenic mouse manifested with multi-organ damage infected with MERS-CoV could be a suitable model for the pathogenic mechanisms study.

Systemic inflammation is believed to be a primary reason for the severe outcome in MERS-CoV infections [14, 24]. Although the transgenic mice model died after MERS-CoV infection with multi-organ damages, it is still uncertain whether these mice are mainly dying from lung damage. In our transgenic mice, aberrant immune response such as elevated IP-10, IFN-γ and IL-17, which are closely related to acute virally-mediated lung injury [39–42], was also a specific manifestation after MERS-CoV infection. So, as to the mechanism of lethal which was due to the virus replication directly or induced by the aberrant inflammatory response need to be further studied. The mechanism of MERS-CoV infection caused death is complex and complicated. Aberrant immune response after MERS-CoV infection may be one of main reasons which need to be further studied. We have added more discussions on this issue in the revised manuscript.

In summary, the transgenic mouse model developed in this study was efficiently infected by MERS-CoV and exhibited severe acute respiratory injury and considerable extrapulmonary organ damage. This model will be useful for studying the pathogenesis of MERS-CoV infection and evaluating the efficacy of MERS vaccines and therapeutic agents.
